# Obstructive Tracheal Necrosis in a Dog Secondary to Smoke Inhalation Injury—Case Report

**DOI:** 10.3389/fvets.2020.00409

**Published:** 2020-07-21

**Authors:** Tommaso Rosati, Jamie M. Burkitt, Katherine D. Watson, Karl E. Jandrey, Laura G. Osborne, Devinn M. Sinnott, Steven E. Epstein

**Affiliations:** ^1^William R. Pritchard Veterinary Medical Teaching Hospital, School of Veterinary Medicine, University of California, Davis, Davis, CA, United States; ^2^Department of Surgical and Radiological Sciences, School of Veterinary Medicine, University of California, Davis, Davis, CA, United States; ^3^California Animal Health and Food Safety Laboratory, School of Veterinary Medicine, University of California, Davis, Davis, CA, United States; ^4^Western Veterinary Specialist and Emergency Centre, Calgary, AB, Canada

**Keywords:** canine, lung injury, delayed neurological signs, anthracosis, upper airway obstruction, tracheal obstruction, airway cast, critical care

## Abstract

A 4-year-old Siberian Husky mix was referred to the emergency service of the University of California Davis Veterinary Medical Teaching Hospital after being found unconscious in a housefire. Upon arrival, the dog was conscious and panting with normal breathing effort. The dog was initially treated with oxygen therapy to minimize the risk of carbon monoxide toxicosis. Progressive agitation with paroxysmal episodes of increased respiratory effort and increased upper airway sounds were noted ~48 h after presentation. Hypoxemia was then documented. Clinical signs continued to progress despite supportive measures, and five days after initial presentation mechanical ventilation was deemed indicated. Following anesthetic induction, endotracheal intubation was performed. Capnography and peak inspiratory pressures recorded on the mechanical ventilator were consistent with airway obstruction. Diffuse intraluminal tracheal obstruction with grossly necrotic tracheal tissue was confirmed using fiber optic tracheoscopy. The patient was humanely euthanized due to grave prognosis. At necropsy, the tracheal lumen was obstructed by sloughed, necrotic tracheal mucosa. This is the first report describing a severe delayed intrathoracic large airway complication secondary to smoke inhalation in a dog.

## Background

Smoke inhalation injury (SII) is a severe complication of smoke exposure in animals and people trapped in confined space in the presence of unintentional fire. The prevalence of SII in small animals is unclear, likely due to high prehospital mortality and the absence of a unified database. It is reported that up to 73% of people with SII develop respiratory failure and ~20% of these develop acute respiratory distress syndrome (1). In people, SII has a multifactorial pathogenesis; respiratory and neurological signs are the main features and can occur acutely or be delayed by days. Upper and lower airway damage and pulmonary parenchymal injury can occur associated with smoke exposure. Acute respiratory signs are often a consequence of direct thermal injury, and the derived soft tissue edema to the supraglottic structures with subsequent airway obstruction (2, 3). Delayed respiratory signs are generally attributed to pulmonary parenchymal injury leading to pulmonary edema or secondary bacterial infection. Lung injury leads to ventilation/perfusion (V/Q) mismatch and subsequently hypoxemia. Ventilation can be impaired by bronchial obstruction secondary to cast deposition (2). It is believed that small animal patients exposed to smoke undergo a clinical course similar to people (4).

To the authors' knowledge, this is the first report describing a severe delayed intrathoracic large airway complication secondary to smoke inhalation in a dog.

## Case Presentation

A 4-year-old, 28.6 kg, male castrated Siberian Husky mix was referred to the William R. Pritchard Veterinary Medical Teaching Hospital (VMTH) at the University of California, Davis, for further management after being trapped in a housefire. The dog was found by firefighters and was reportedly unconscious at the time of rescue but improved with supplemental flow-by oxygen therapy provided on site. The dog was subsequently presented to a local veterinary clinic where it was conscious but anxious and tachypneic. The referring veterinarian continued to provide flow-by oxygen therapy, and also administered a 500 ml bolus of intravenous (IV) crystalloid fluid. The dog was transported to the VMTH for 24 h care while receiving supplemental oxygen therapy.

On presentation to the emergency department, the dog was quiet, alert, responsive, and persistently panting. Thoracic auscultation revealed diffusely increased lung sounds with mild referred stertor. Copious serous discharge was present from both nostrils. The heart rate was 128 beats per minute and the remaining perfusion parameters (mucous membrane color, capillary refill time, pulse quality, extremity temperature) were deemed adequate. The rectal temperature was 99.8°F (37.6°C). The whole haircoat was covered in ash and a strong smell of smoke was appreciable. There was no evidence of burn injury over the dog's body surface. Oral cavity inspection revealed the presence of soot material over the tongue and teeth with no ulceration or soft tissue edema noted.

**Figure 1:**
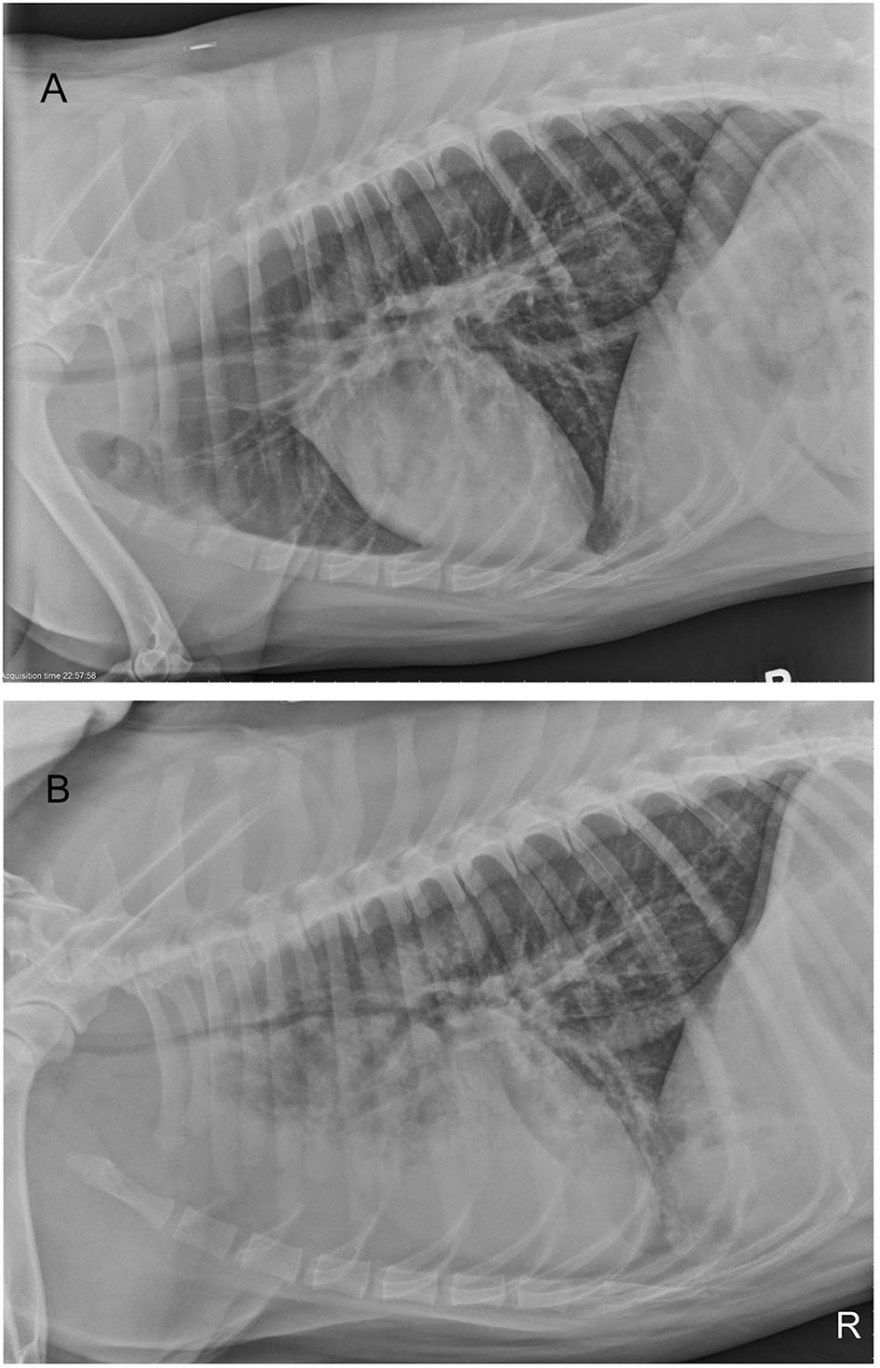
Right lateral thoracic radiographs of a 4-year old Siberian Husky mix with smoke inhalation injury **(A)** radiograph obtained on presentation and **(B)** radiograph performed 4 days after presentation. Progressive tracheal narrowing and progressive, ventrally dependent interstitial to alveolar pulmonary pattern are present. Diffuse interstitial to bronchial pattern is present on both studies.

At presentation (Day 1), an arterial blood gas on room air revealed that pulmonary function was within normal limits with PaO_2_ 91 mmHg and PaCO_2_ 31 mmHg with a normal pH and Lactate (7.40 and 1.1 mmol/L, respectively). Thoracic radiographs showed mild tracheal narrowing and a ventrally distributed interstitial to alveolar pulmonary pattern (Figure 1A). Throughout the remainder of the lung fields was a mild, diffuse bronchointerstitial pattern. Complete blood count (CBC) revealed mild leukocytosis (total white blood cell count 18,820/μl, reference interval 6,000-13,000/μl), characterized by neutrophilia (neutrophil count 15,621/μl, reference interval 3,000–10,500/μl), with mild left shift (band neutrophils 753/μl).

The dog was hospitalized for further monitoring and care. Supplemental oxygen was provided during the 1st hour after arrival via a face mask to hasten elimination of any persistent carbon monoxide (CO); co-oximetry for carboxyhemoglobin measurement was not available. Intermittent sterile water nebulization, ampicillin-sulbactam (Pfizer, New York, NY, USA; 30 mg/kg IV every 8 h), intravenous fluid therapy with lactated Ringer's solution (Baxter Healthcare, Deerfield, IL, USA) at 50 ml/h IV (1.75 ml/kg/h) and ondansetron (Mylan, Canonsburg, PA, USA; 0.5 mg/kg IV every 12 h) were also provided. The patient remained anxious and tachypneic developing progressively increased lung sounds and audible stertor with occasional coughing and exaggerated swallowing over the first 12–24 h of hospitalization. An anti-inflammatory dose of dexamethasone sodium phosphate (VetOne, Boise, ID, USA; 0.05 mg/kg IV) was given within the first 24 h of presentation and continued every 24 h. To control anxiety, an IV continuous rate infusion of dexmedetomidine (Zoetis, Parsippany, NJ, USA) was started (range, 0.5–2 μg/kg/h) and intermittent boluses of butorphanol (Zoetis, Parsippany, NJ, USA; 0.2 mg/kg IV) were administered as required.

During days 2 and 3, progressive tachypnea and anxiety were noted. Thoracic auscultation revealed worsening referred upper airway noises. Intermittently the dog would become extremely agitated and would show signs of respiratory distress with marked increased respiratory effort and worsening tachypnea. Respiratory distress episodes were treated with sedation (butorphanol 0.2 mg/kg IV and acepromazine (VetOne, Boise, ID, USA) 0.01 mg/kg IV as needed) and flow-by oxygen therapy. Trazodone (Zydus, Pennington, NJ, USA; 75 mg by mouth every 12 h) was added, to assist with anxiolysis. On day 4, thoracic radiographs were repeated (Figure 1B). Progressive ventrally distributed alveolar pulmonary pattern and worsening tracheal and bronchial narrowing were reported. An arterial blood gas on room air confirmed hypoxemia (PaO_2_ 57 mmHg) and hyperventilation (PaCO_2_ 28 mmHg). A CBC reported a white blood cell count within laboratory reference interval (total white blood cell count 12,030/μl, reference interval 6,000–13,000/μl) with no band neutrophils. Continuous oxygen supplementation was re-instituted via nasal cannula at 178 ml/kg/min. Despite oxygen supplementation the dog remained agitated and an IV continuous rate infusion of butorphanol (range 0.05–0.1 mg/kg/h) was initiated. On day 5, persistent hypoxemia (PaO_2_ 61 mmHg) with PaCO_2_ 32 mmHg was reported while receiving oxygen supplementation at 178 ml/kg/min via nasal cannula. Heated, humidified oxygen therapy (Precision Flow® Hi-VNI, Vapotherm Inc., Exeter, USA) was thus instituted (range 258–464 ml/kg/min) at a temperature of 37°C and FiO_2_ of 1.0 (100% oxygen). Additionally, enrofloxacin (Bayer Healthcare LLC, Shawnee Mission, KS, USA) (10 mg/kg IV every 24 h) was initiated. To treat potential discomfort IV sufentanil (Akorn inc, Lake Forest, IL, USA) CRI (range, 0.001–0.005 μg/kg/min) was started. Progressive abdominal distention was noted and a nasogastric tube was placed in order to facilitate gas removal from the stomach. Despite therapeutic adjustments, tachypnea with increased respiratory effort with intermittent distress continued. Due to limited duration of distress episodes and to financial considerations mechanical ventilation was discussed with clients but not pursued. During paroxysmal episodes of apparent agitation and distress, several arterial blood gas measurements were performed, and worsening of hypoxemia or hypoventilation was never documented (Table 1). Serial neurological examinations were performed because of the paroxysmal episodes and no significant abnormalities were definitively identified. Loss of consciousness or tonic-clonic muscle contraction were never documented. It was considered possible that the paroxysmal episodes of agitation were atypical seizure activity due to carbon monoxide toxicosis; thus, phenobarbital (West-Ward, Eatontown, NJ, USA) (4 mg/kg IV every 12 h) was started.

**Table 1:** Selected values from arterial blood gases obtained during the 5-day hospital stay.

**Day**	**1**	**3**	**5**	**5**	
**Event**	**At admission**	**During respiratory distress event**	**Before anesthesia induction**	**After intubation**	
			**O**_**2**_ **supplementation via HFOT**	**On the ventilator**	**Reference Interval**
Inspired oxygen	21%	21%	13 L/min	100%	
pH	7.41	7.47	7.41	6.96	7.35–7.46
PaCO_2_	31	28	40	125	31–43 mmHg
PaO_2_	91	57	64	93	85–100 mmHg
SBE	−4.5	−2.5	1.1	−4.7	−4–4 mmol/L
Lactate	1.1	0.4	1.1	1.5	<2 mmol/L
PaO_2_/FiO_2_ ratio	433	271	N/A	93	400–500

*HFOT, high-flow oxygen therapy; SBE, standard base excess; N/A, not applicable*.

On day 6, tachypnea with progressively worsening respiratory effort and persistent hypoxemia (PaO_2_ 64 mmHg) despite oxygen supplementation (13 L/min) were still present. PaCO_2_ was 40 mmHg. Due to concern for excessive work of breathing and impending muscle fatigue, mechanical ventilation was recommended. General anesthesia was induced with IV propofol (Zoetis, Parsippany, NJ, USA) titrated to effect (total dose 3.14 mg/kg). During the induction phase the dog's cardiovascular function was monitored via ECG and continuous invasive blood pressure monitoring, and no significant abnormalities were recorded. Endotracheal tube (ETT) positioning was estimated by evaluating the distance between the dog's nasal planum and the thoracic inlet. The dog's trachea was intubated with a size 10 mm inner diameter ETT. Correct positioning was confirmed by direct visualization of the ETT passing into the larynx during intubation. After intubation, the ETT was secured to the dog's muzzle and ETT cuff was inflated. Upon connection to the mechanical ventilator circuit and to a capnograph, absent end tidal CO_2_ was noted. Initial ventilator settings were pressure controlled ventilation with a peak inspiratory pressure of 12 cmH_2_O and a positive end expiratory pressure of 5 cmH_2_O resulting in a 0 ml tidal volume delivery. Peak inspiratory pressure was incrementally increased to 30 cmH_2_O with a resulting tidal volume of 2–5 ml recorded by the ventilator and no end-tidal CO_2_ detected. In order to re-confirm correct ETT position, the ETT was removed and a 9.5 mm inner diameter ETT was used to re-intubate the trachea. A fiber optic bronchoscope (PortaView® LF-TP, Olympus Corporation) was used to visualize the tracheal lumen. Intraluminal obstruction secondary to collapse of the dorsal portion of the intrathoracic trachea was directly visualized and confirmed to extend as far distal as the carina. The tracheal mucosa of the collapsing area appeared grossly ulcerated and necrotic. Multiple ventilator settings and modes including airway pressure release ventilation and manual ventilation were attempted and unsuccessful at delivering an adequate tidal volume. An arterial blood gas was performed while 100% oxygen was delivered via the mechanical ventilator, PaO_2_ was 93 mmHg with a PaO_2_:FiO_2_ (ratio of arterial oxygen partial pressure to fractional inspired oxygen) of 93. Severe hypoventilation (PaCO_2_ 125 mmHg) with marked respiratory acidosis (pH 6.96) was simultaneously documented. Grave prognosis was discussed with the owners, who elected humane euthanasia of the dog.

**Figure 2:**
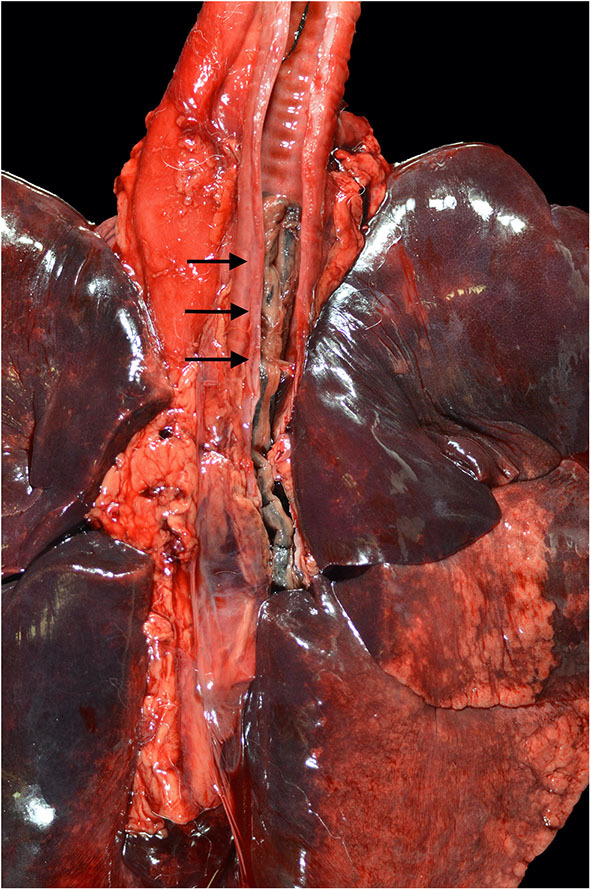
Necropsy findings of a 4-year-old Siberian Husky mix with smoke inhalation after being in a housefire. Severe, diffuse tracheal mucosal necrosis, and subsequent intraluminal tracheal obstruction can be observed (black arrows). Diffuse pulmonary parenchymal edema and diffuse deposition of particulate were described.

**Figure 3:**
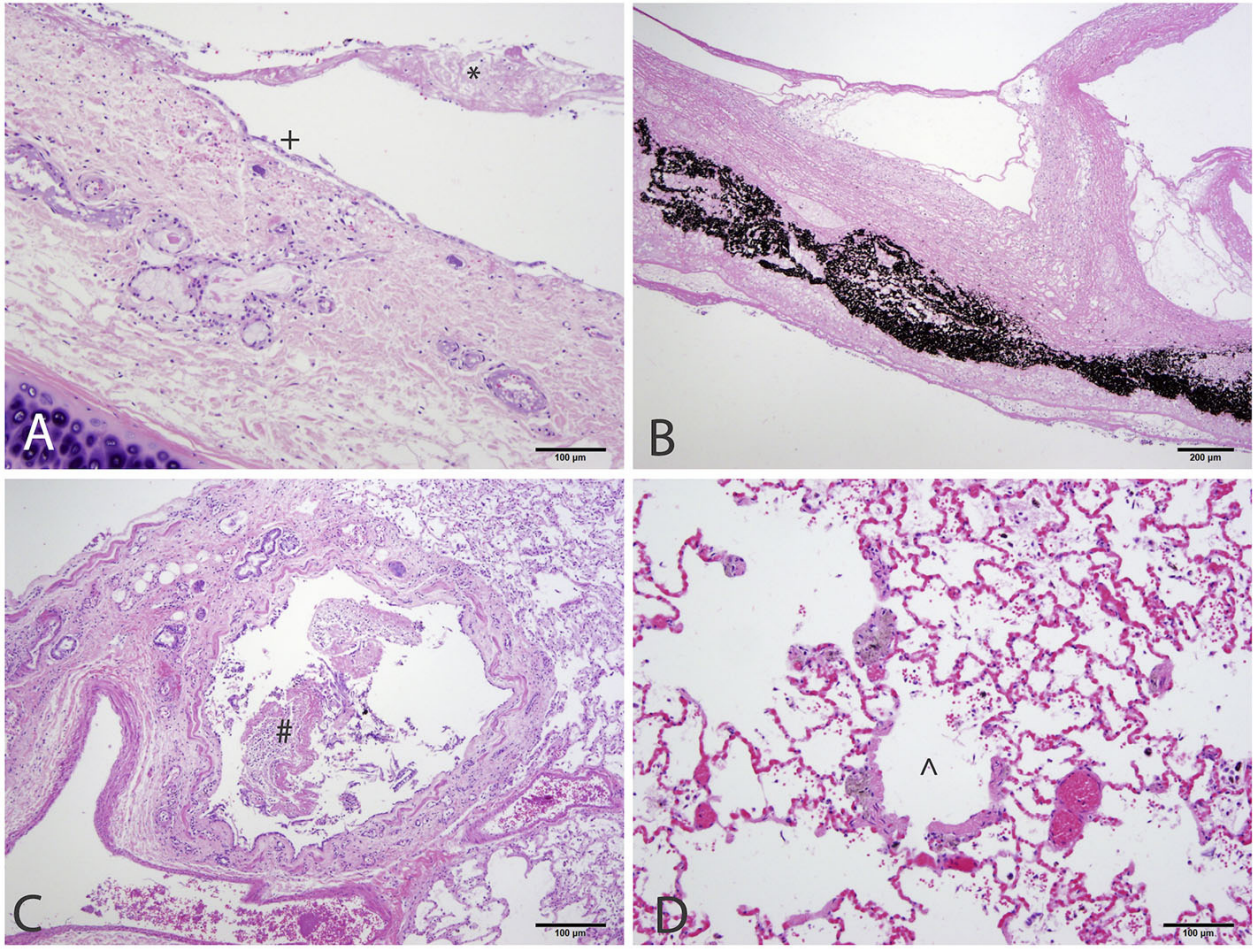
Histology of the respiratory tract of a 4-year-old Siberian Husky mix with smoke inhalation after being in a housefire. **(A)** Trachea with attenuated epithelium (+) with streaming necrotic material extending from the ulcerated mucosa (*). **(B)** The lumen was obstructed by necrotic tissue admixed with fibrin and black particulate matter (interpreted as soot). **(C)** Bronchi have similarly attenuated to ulcerated mucosa with necrotic and cellular debris within the lumen. **(D)** Bronchiolar smooth muscle is expanded by granular, dark brown to black pigment (interpreted as carbon particles). Alveoli are variably ruptured forming coalescing airspaces (alveolar emphysema).

At necropsy the mucosal lining of the proximal and mid trachea were sloughed into the distal tracheal lumen forming a coagulum of necrotic tissue that occluded the airway. There was no overt evidence of tracheal cartilage deformation. Predominantly the cranioventral lung lobes were heavy, wet, and dark red (Figure 2). On cut-section they oozed serosanguineous fluid. Histologically the remaining tracheal mucosal surface was necrotic or attenuated (Figure 3A). The intraluminal coagulum was composed of necrotic tissue, fibrin, and black particulate matter (interpreted as soot, Figure 3B). Bronchi had similarly attenuated to necrotic epithelium with intraluminal necrotic and cellular debris (Figure 3C). Bronchiolar smooth muscle was expanded by fine, black to dark brown pigment (interpreted as carbon particles or anthracosis). Alveoli were variably hyperinflated and confluent with shortened alveolar septa with blunt, clubbed ends (alveolar emphysema, Figure 3D).

## Discussion

This is the first report of delayed airway obstruction secondary to extensive mucosal sloughing and tracheobronchial cast formation after smoke inhalation in a dog. The progressive respiratory signs reported in this dog were likely a consequence of pulmonary parenchymal injury and airway obstruction.

The composition of smoke is unique for each fire and depends largely on the material burned and the availability of oxygen. The major components of smoke that can lead to SII are heat, super-heated particles, airborne irritant, and toxic gases (5). Multiple airborne irritants have been identified in smoke, but the clinical effect is similar in most cases. Super-heated particles, also known as soot, are particulates that contribute significantly to SII by causing direct thermal damage to the airway mucosa. Additionally, soot particles can function as a carrier to the alveoli for a variety of toxins (5). Toxic gas inhalation can lead to acute and delayed neurological signs (5).

### Tracheal Necrosis

During smoke exposure, significant heat dissipation occurs within the larynx and consequently direct thermal injury to the tracheobronchial system is unusual (6). In the case presented here, it is likely that clinically significant pharyngeal and laryngeal damage did not occur due to lack of heat exposure. Lower airway injury is largely secondary to chemical inhalation causing direct damage as well as neurogenic inflammation (7). Halogen acids, formaldehyde, and unsaturated aldehydes are common respiratory irritants present in smoke (7). Chemical irritation leads to respiratory mucosal damage and sloughing into the airway, as well as a profound induction of the victim's inflammatory response. The airways are innervated by vasomotor and sensory nerve endings that, once stimulated by the chemical injury, lead to production of neuropeptides (8). Neuropeptides such as substance P and calcitonin gene-related peptide induce bronchoconstriction, nitric oxide production, and the generation of reactive oxygen species (ROS) (9). Additionally, neuropeptides can promote neutrophil chemotaxis and consequently increase the host inflammatory response (9).

Progressive tracheal damage has been documented in a canine laboratory model via inhalation of unheated smoke produced by incomplete combustion of sawdust, wheat grass, and cotton fibers. In that experimental study, microscopic examination of the trachea 2 h after smoke exposure showed wall necrosis, mucosal and submucosal hemorrhage, and edema, with neutrophilic infiltration. Tracheal damage progressively worsened over the course of the first 24 h. The chemical composition of smoke produced in this laboratory model might not be representative of a household scenario, although the results of this study highlight the relevance of airway damage secondary to inhalation of soot and airborne irritants even without excessive heat (10). The case reported here is the first clinical report of mucosal sloughing and tracheobronchial cast formation in a dog due to SII. To the authors' knowledge tracheal necrosis secondary to SII has never been reported in people. In this dog, progressive tracheal narrowing was noted on thoracic radiographs. Based on necropsy findings, the reported tracheal necrosis likely occurred due to inhalation of superheated particles and airborne irritants. Interestingly, hypercapnia as a consequence of airway obstruction was not documented prior to anesthesia induction. However, considering the pulmonary parenchymal irritation caused by inhalation of irritant gases, increased minute ventilation and hypocapnia would have been expected (11). Thus, the documented normocapnia may be construed as relative hypoventilation secondary to upper airway obstruction. It is likely that while the dog was conscious, it was able to generate subatmospheric pleural pressures adequate to maintain a patent tracheal lumen and normocapnia. It is theorized that after anesthetic induction, application of positive pressure ventilation in this dog led to removal of negative pleural pressure that had been maintaining tracheal luminal patency. Additionally, it is considered possible that intubation and the application of positive pressure ventilation worsened the ongoing obstructive process by dislodging a previously formed mucosal eschar. The peak inspiratory pressures and positive end expiratory pressure applied were not sufficient to maintain airway patency and to achieve adequate gas exchange. Pressures needed to maintain patency of the trachea would likely have been unsafe for the lung, resulting in barotrauma to the pulmonary parenchyma.

As reported on necropsy, a large portion of the tracheal mucosa had sloughed. On retrospective evaluation, it is likely that the level of pain experienced by the dog was higher than assessed during clinical evaluation. Therefore, early administration of a combination of analgesics such as full mu agonist opioids and ketamine should be considered for future cases.

Fiber optic bronchoscopy has been advocated in people with suspected SII because it allows direct airway visualization and assessment of tissue damage such as erythema and epithelial sloughing that can occur from thermal damage or chemical inhalation (2, 12). Additionally, during bronchoscopy the clinician can decide whether the patient is likely to require intubation to maintain airway patency (13). Unlike in people, bronchoscopy in small animals requires general anesthesia. Due to the invasiveness of the procedure, bronchoscopy might not be feasible in all veterinary patients. It is possible that airway obstructive disease would have been identified earlier in this dog if tracheoscopy had been performed sooner; however, this would likely have precipitated the need for mechanical ventilation earlier. It seems unlikely that clinical course would have changed with earlier tracheoscopy in this case.

Considering the clinical scenario, further interventions were not considered safe and financially feasible for treatment of this dog. However, speculating treatment options, tracheal intubation could have been attempted with a significantly smaller size ETT. Passing through the obstructive intraluminal material may have allowed an alternative ventilatory modality such as jet ventilation. A portion of the sloughed necrotic material could have been removed from the tracheal lumen by forceps retraction via fiber optic bronchoscopy. Considering the extent of the tracheal damage recorded in this patient, tracheal stenting was not considered a viable option; however, this treatment option could be considered for future cases with evidence of tracheal narrowing. Ultimately, although not readily available in veterinary medicine, extracorporeal membrane oxygenation (ECMO) techniques might constitute a treatment option in the future.

### Lung Parenchyma Injury

In the case reported here, diffuse pulmonary parenchymal injury was documented on necropsy. In SII victims, pulmonary parenchymal damage is usually delayed. Alveolar damage in SII is characterized by alveolar collapse due to increased pulmonary transvascular fluid flux and the formation of pulmonary edema (14). In a sheep model, the degree of fluid flux was proportional to the duration of smoke exposure (15). Transvascular fluid shift is promoted by increased microvascular pressure and permeability to proteins (14). The pro-coagulant state associated with SII leads to massive alveolar fibrin deposition and subsequent worsening of V/Q mismatch (16). Additionally, fibrin deposition directly inhibits surfactant production, which exacerbates pulmonary atelectasis due to increased surface tension within the alveolar sacs (17). During SII, ROS, nitric oxide, and reactive nitrogen species are produced in the respiratory system. The overproduction of nitric oxide has been reported to inhibit physiologic hypoxic pulmonary vasoconstriction and consequently to increase V/Q mismatch and worsen hypoxemia (18).

Pulmonary parenchymal injury is further exacerbated by neutrophils becoming trapped in the pulmonary capillary bed (19). After adhering to capillary endothelial cells, activated neutrophils cause direct pulmonary parenchymal damage by releasing proteases and ROS (20).

In the case reported here, from the time the first set of thoracic radiographs was obtained, the dog received empiric antimicrobial therapy. Despite the fact that prophylactic antimicrobial use is not indicated in SII, the ventral distribution of the pulmonary interstitial pattern and the dog's history of unconsciousness at the fire scene led clinicians to believe the dog may have had aspiration pneumonia complicating the smoke inhalation. Alternately, it is possible that the pulmonary pattern represented changes due to SII alone. In a retrospective case series of dogs exposed to smoke, the most common radiographic findings were an alveolar or interstitial pattern, though the authors did not identify a consistent pattern of distribution (21). Bacterial culture and cytological evaluation of an airway sample (from tracheal wash or bronchoalveolar lavage) could have been useful to distinguish between an infected and a purely inflammatory process. In human burn victims with concurrent SII, the incidence of pneumonia is reported to be two- to four-fold higher than in patients without SII (22, 23). The higher incidence of pneumonia in that patient population is suspected to be secondary to destruction of airway epithelium and dysfunction of the mucociliary apparatus. As previously mentioned, in this case a sample for culture and susceptibility from an airway was not obtained due to concern for patient stability. A tracheal wash was planned to be performed after intubation for mechanical ventilation, but was aborted since the dog was euthanized. On day 5, based on the absence of clinical improvement, empiric antimicrobial escalation with enrofloxacin was considered reasonable. The authors acknowledge that fluoroquinolones should be reserved for patients with documented susceptible bacterial infection or for critically ill animals in order to reduce the risk of bacterial resistance. No evidence of bacterial infection was noted at necropsy, though no cultures were performed of post-mortem pulmonary tissues.

### Delayed Neurological Signs

Delayed neurological signs following SII have been previously documented in the veterinary literature. In a case report from 2003, a 1-year-old dog developed stupor and non-ambulatory tetraparesis 4 days after exposure to smoke in a house fire (24). Full recovery of the neurological function was reported in ~1 week. In another retrospective case series, 11 dogs were evaluated after smoke exposure. Acute, delayed neurologic signs developed in five of the dogs (25).

Acute neurological signs after SII have been primarily attributed to the presence of inhaled toxicants such as CO. CO toxicosis is a well-recognized risk factor for development of neurologic complications in smoke inhalation patients (26–28). In order to minimize risk of CO toxicosis, the dog reported here was initially treated with oxygen therapy via nasal cannula. CO half-life is ~320 min in patients breathing room air but can be reduced to as little as 70 min in patients breathing 100% oxygen (29). Carbon monoxide binds to hemoglobin to form carboxyhemoglobin. CO affinity for hemoglobin is 200–250 times greater than oxygen's affinity for hemoglobin, and carboxyhemoglobin reduces hemoglobin's oxygen carrying capacity and thereby leads to tissue hypoxia (29). Other mechanisms of intracellular toxicity secondary to CO have been described. Carbon monoxide inhibits cytochrome oxidase systems at the level of the mitochondria and thus inhibits the cell's ability to utilize oxygen. Additionally, inhibition of the cytochrome-c oxidase leads to mitochondrial oxidative stress due to ROS production, which causes neuronal necrosis and apoptosis (30). Neurological injury is also promoted by additional inflammatory pathways promoted by CO independent of hypoxia (30).

In the case reported here, it was unclear whether the dog's paroxysmal episodes were neurologic, respiratory, or behavioral in origin. Electroencephalography could have been used to identify abnormal cortical electrical activity to definitively determine whether the paroxysms were seizure activity. The addition of anticonvulsant therapy did not improve the dog's clinical signs.

This case report describes a severe respiratory complication in a dog secondary to SII. Delayed obstructive large airway disease should be considered a differential diagnosis in small animal SII victims showing respiratory signs.

## Data Availability Statement

The raw data supporting the conclusions of this article will be made available by the authors, without undue reservation.

## Ethics Statement

The dog detailed in the case report presented as a patient to the William R. Pritchard Veterinary Medical Teaching Hospital in Davis, CA. The clients signed a consent form to permit hospitalization and treatment. Additional consent was obtained for anonymized necropsy and post-mortem samples for research purposes.

## Author Contributions

TR, JB, and KW were involved in the preparation of the manuscript, and all authors contributed to the manuscript revision and approved the submitted version.

## Conflict of Interest

The authors declare that the research was conducted in the absence of any commercial or financial relationships that could be construed as a potential conflict of interest.
